# High dietary advanced glycation end products are associated with poorer spatial learning and accelerated Aβ deposition in an Alzheimer mouse model

**DOI:** 10.1111/acel.12436

**Published:** 2016-01-19

**Authors:** Irit Lubitz, Jan Ricny, Dana Atrakchi‐Baranes, Chen Shemesh, Efrat Kravitz, Sigal Liraz‐Zaltsman, Anna Maksin‐Matveev, Itzik Cooper, Avshalom Leibowitz, Jaime Uribarri, James Schmeidler, Weijing Cai, Zdena Kristofikova, Daniela Ripova, Derek LeRoith, Michal Schnaider‐Beeri

**Affiliations:** ^1^ The Joseph Sagol Neuroscience Center Tel HashomerRamat Gan52621Israel; ^2^ Department of Biochemistry and Brain PathophysiologyNational Institute of Mental HealthTopolova 748Klecany250 67Czech Republic; ^3^ Department of Internal Medicine D and Hypertension UnitSheba Medical CenterRamat Gan52621Israel; ^4^ Department of PsychiatryMount Sinai School of MedicineNew YorkNY10029USA; ^5^ Department of MedicineIcahn School of Medicine at Mt SinaiNew YorkNY10029USA

**Keywords:** advanced glycation end product, Alzheimer's disease, Aβ, blood–brain barrier, receptor for advanced glycation end product, Tg2576

## Abstract

There is growing evidence of the involvement of advanced glycation end products (AGEs) in the pathogenesis of neurodegenerative processes including Alzheimer's disease (AD) and their function as a seed for the aggregation of Aβ, a hallmark feature of AD. AGEs are formed endogenously and exogenously during heating and irradiation of foods. We here examined the effect of a diet high in AGEs in the context of an irradiated diet on memory, insoluble Aβ_42_, AGEs levels in hippocampus, on expression of the receptor for AGEs (RAGE), and on oxidative stress in the vasculature. We found that AD‐like model mice on high‐AGE diet due to irradiation had significantly poorer memory, higher hippocampal levels of insoluble Aβ_42_ and AGEs as well as higher levels of oxidative stress on vascular walls, compared to littermates fed an isocaloric diet. These differences were not due to weight gain. The data were further supported by the overexpression of RAGE, which binds to Aβ_42_ and regulates its transport across the blood–brain barrier, suggesting a mediating pathway. Because exposure to AGEs can be diminished, these insights provide an important simple noninvasive potential therapeutic strategy for alleviating a major lifestyle‐linked disease epidemic.

## Introduction

Advanced glycation end products (AGEs) are generated from multiple sources and mechanisms (Uribarri *et al*., 2010). Chronic accumulation of AGEs is accelerated with aging and with aging‐related diseases such as diabetes, hyperlipidemia, and renal disease (Cho *et al*., 2009). Advanced glycation end products AGEs may accumulate in vascular tissues, thickening and stiffening vascular walls, and increasing the risk of hypertension (Singh *et al*., 2001; Goh & Cooper, 2008). Higher levels of AGEs are associated with cognitive impairment in people with cerebrovascular disease, suggesting a relationship between AGEs and vascular dementia (Southern *et al*., 2007). Moreover, there is evidence of AGEs involvement in the pathogenesis of neurodegenerative processes including Alzheimer's disease (AD) (Grossman, 2003). We recently reported that elevated serum AGEs level is associated with a faster rate of cognitive decline (Beeri *et al*., 2011; West *et al*., 2014).

There is a growing body of evidence indicating that the interaction of AGEs with their receptor [receptor for AGEs (RAGE)] elicits oxidative stress and inflammatory reactions. In addition, RAGE has been shown to function as a signal‐transducing cell surface receptor for Aβ_42_ to induce reactive oxygen species (ROS) (Arancio *et al*., 2004; Chaney *et al*., 2005). Moreover, RAGE is increased in brains of AD patients, (Yan *et al*., 1996; Lue *et al*., 2001) and has a role in the regulation of Aβ transport across the blood–brain barrier (BBB) (Lue *et al*., 2001; Askarova *et al*., 2011).

Advanced glycation end products AGEs are long known to form in foods during heating (Lee *et al*., 1981; O'Brien & Morrissey, 1989; Koschinsky *et al*., 1997). There are two well‐characterized compounds, *N*‐carboxy methyl‐lysine (CML) and methyl‐glyoxal (MG), derivatives of glucose–protein or glucose–lipid interactions, which serve as markers for AGEs (Dyer *et al*., 1992; Vlassara & Palace, 2002). A positive correlation is shown between the amount of AGEs consumed and that found in the circulation (Koschinsky *et al*., 1997; He *et al*., 1999). Consistent with our findings of high serum MG association with faster cognitive decline, we have shown that in the elderly, consumption of high dietary AGEs is associated with a faster rate of cognitive decline (Cai *et al*., 2014). Food‐derived AGEs can mimic the actions of endogenously formed AGEs and can cause oxidative stress leading to diabetes complications (Yamagishi *et al*., 2012; Cai *et al*., 2014).

The goal of this study was to examine the effect of a irradiation diet (henceforth high‐AGE diet) on memory, AD neuropathology and RAGE expression in the brain of a mouse model of AD (Tg2576) that overproduce Aβ. Tg2576 and their littermates were fed with a high‐AGE diet or nonirradiation diet (henceforth regular AGE diet), for 8 months. We hypothesized that treatment with a high‐AGE diet due to irradiation would induce poor memory and increased AD‐like neuropathology mediated by RAGE. Indeed, mice receiving a high‐AGE diet had poorer memory and increased arterial oxidative stress than their littermates on regular diet. Furthermore, the Tg2576 mice on high‐AGE diet showed higher amount of AGEs in the hippocampus, and the level of insoluble Aβ_42_ was associated with AGEs excretion in urine.

## Results

### General

**Figure 1:**
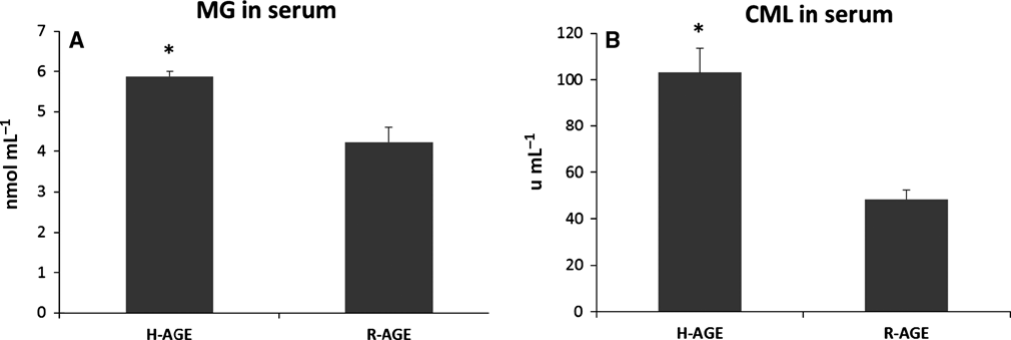
ELISA analysis of AGES levels in serum, after 1 month on the diet, MG (A) and CML (B). Mean ± SEM, *P < 0.001, *n* = 9 per group.

In a preliminary experiment of one month, we showed a significantly higher serum level of CML and MG in mice fed H‐AGE diet (Fig. 1). When comparing mice groups on H‐AGE and R‐AGE diet in the long‐term experiment*,* there were no baseline differences in body weight, serum fasting glucose, CML, or MG. At the end of the study (at 11 months of age*,* Table 1), the groups did not differ in weight gain, serum fasting glucose or food intake (measured in metabolic cages). Tg2576 mice on H‐AGE diet had significantly higher MG and nominally higher CML (Table 2). Open field testing did not show anxiety or motor difficulties in any of the groups.

**Table 1:** Characteristics of animals

	WT H‐AGE	WT R‐AGE	Tg H‐AGE	Tg R‐AGE
Δ Weight gain (g)	7.9 ± 0.18	8.74 ± 0.17	8.74 ± 0.22	7.6 ± 0.24
End weight (g)	37.1 ± 0.17	38.5 ± 0.19	34.3 ± 0.22	33.4 ± 0.24
Fasting glucose baseline (mg dL^−1^)	107.5 ± 2.8	115 ± 7.4	118.7 ± 8	110 ± 6.5
Fasting glucose at end (mg dL^−1^)	140 ± 5.3	139 ± 5.9	146 ± 11.4	136.5 ± 6.7

Data are means ± SEM, ns.

**Table 2:** Pearson correlation of insoluble Aβ42 in hippocampuses of Tg2576 mice with levels of CML and MG detected in urine

	Correlations	
	CML	MG
Insoluble Aβ_42_		
H‐AGE, *N* = 10	0.619^a^	0.632^b^
R‐AGE, *N* = 17	−0.019	0.067

a Pearson Correlation is significant at the 0.05 leval (two‐tailed).

b *P*‐value = 0.057.

#### Tg2576 mice on H‐AGE diet show learning impairment

**Figure 2:**
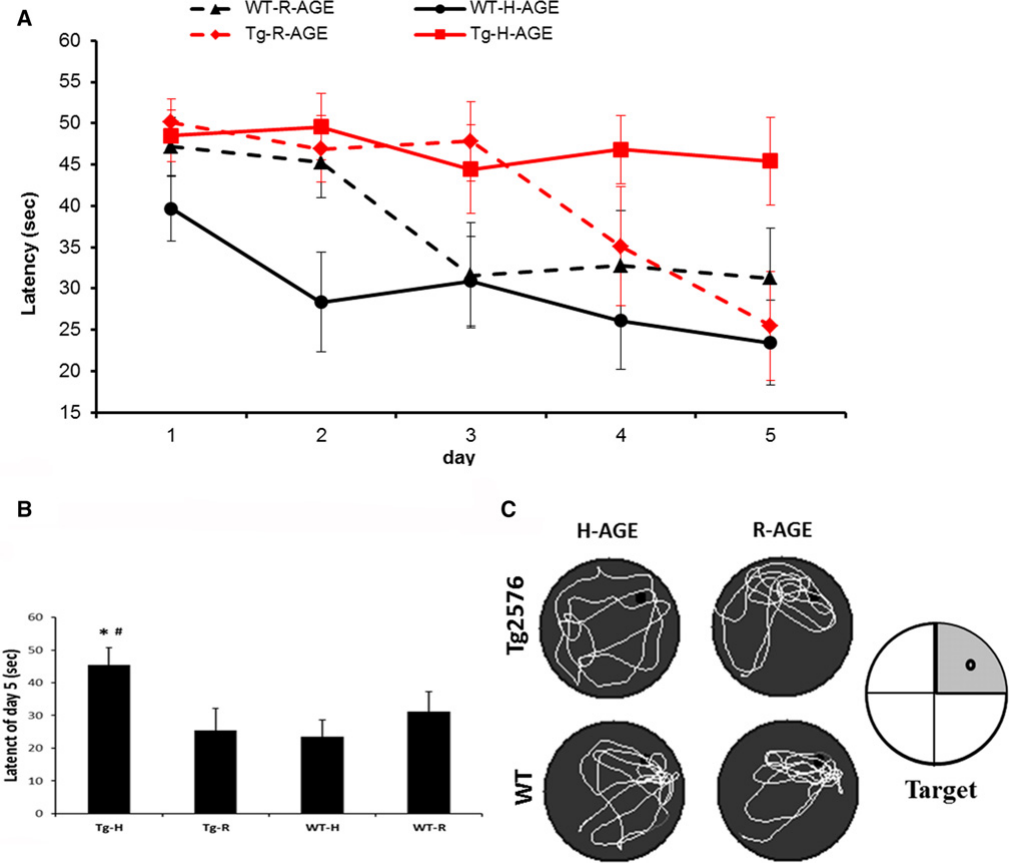
Exacerbation of learning and memory deficit in Tg2576 mice, on high‐AGE diet. (A–C) Morris water maze test at 11 month. Escape latencies in hidden‐platform trails (*n* = 10 per group). *P* < 0.05 for genotype *× *day *× *diet (A) latency to locate platform at day 5 *P < 0.05 vs. Tg‐R; ^#^ *P* < 0.01 vs. WT‐H (B) Swimming pattern, black dot indicates the area where the platform was located (NS). (C) Data are mean ± SEM.

All mice were tested for spatial learning and memory in the Morris Water Maze (MWM), at the age of 11 months. Swimming speed during each of the 5 days of the acquisition phase (hidden‐platform training) did not significantly differ between the groups (nonsignificant repeated‐measures analysis of variance (anova) genotype main effect, diet main effect, and genotype × diet interaction effect; data not shown). Wild‐type (WT) mice on both diets showed learning of the task, as escape latencies to the platform decreased during the 5 days of the acquisition phase. Tg2576 mice showed impaired learning of the task (Fig. 2A), as expected (Hsiao *et al*., 1996). Tg2576 mice on R‐AGE diet did learn the task, but the learning was substantially delayed as the time to locate the hidden platform did not decrease during days 1–3 and then decreased at days 4 and 5 of the test to a level similar to those of WT mice. Tg2576 mice on H‐AGE diet were the only group that did not learn the hidden‐platform location, reflected in consistently long escape latencies during the 5 days of hidden‐platform training. Repeated‐measures ANOVA showed a significant difference in escape latencies between the two genotypes (*F*_1,31_=5.66, *P *=* *0.024). In addition, genotype had a significant effect on the interaction of diet with learning (day *× *genotype *× *diet interaction: *F*_3.765,116.72_ = 2.511, *P *=* *0.049), indicating that the deleterious effect of H‐AGE diet on learning was stronger in Tg2576 mice than the WT. Finally, considering day 5 alone, the last day of hidden‐platform training, Tg2576 mice on H‐AGE diet had significantly longer escape latencies than Tg2576 mice on R‐AGE diet and WT mice on H‐AGE diets (*P* = 0.04 and *P* = 0.01, respectively, Fisher's PLSD *post hoc* test).

For the memory test (probe trial), the platform was removed and mice were allowed to explore the pool for 60 s. The target quadrant (TQ) is the quadrant in which the platform was formerly located. None of the groups showed a significant bias toward the TQ (nonsignificant two‐way anova for time spent in TQ, and for each group *t*‐test for time in TQ compared to the average in other quadrants).

#### Chronic intake of AGEs is associated with higher levels of peripheral AGEs and insoluble Aβ_42_

In the Tg2576 H‐AGE diet group (but not in the R‐AGE diet group), the correlation of Aβ42 in the hippocampus with urine CML approached significance (*r* = 0.619; *P* = 0.057) and was significant with MG (*r* = 0.632; *P* = 0.050; Table 2). At the age of 11 months, the WT groups did not develop detectable levels of Aβ42 (Table 2).

#### Effect of H‐AGE on oxidative stress

**Figure 3:**
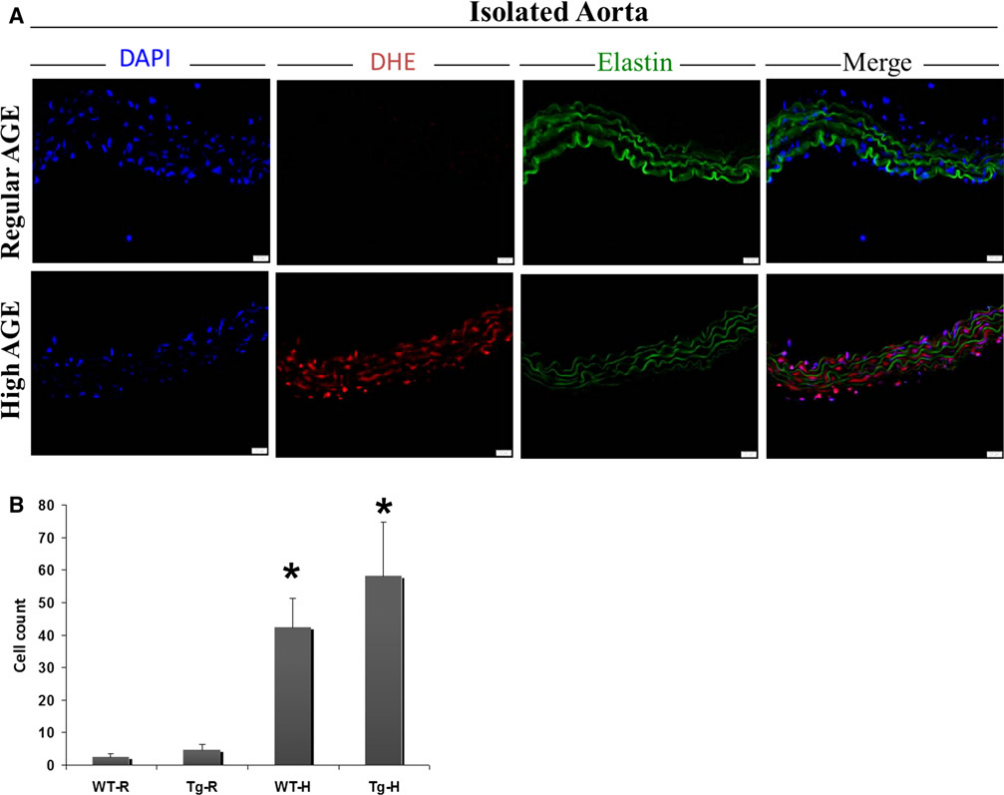
*In situ* detection of superoxide production with dihydroethidium (DHE) in isolated aorta. (A) DHE staining in Tg2576 mice on H‐AGE diet compared to R‐AGE diet (*n* = 10 per group). (B) Calculation of cell staining with DHE in vessels from the different diets, combined red and blue fluorescence, was quantified in three sections per mice. *P =* *0.01 for Tg‐H vs. Tg‐R and for WT‐H vs. WT‐R. Scale bar 20 μm.

We examined the levels of $O_{2}^{\cdot -}$ formation evaluated by the oxidative fluorescence dye DHE in the aorta of the four groups of mice at 11 months (Fig. 3A). ROS levels, as demonstrated by red staining DHE, were elevated in Tg2576 and WT on H‐AGE diet compared to R‐AGE diet. The amount of ROS in Tg2576 H‐AGE was the highest (Fig. 3B). The increase in superoxide production suggested that AGEs, due to irradiation are able to induce/augment oxidative stress in the vasculature.

#### Increased expression of RAGE in cortex of 11‐month‐old mice fed high‐AGE diet

**Figure 4:**
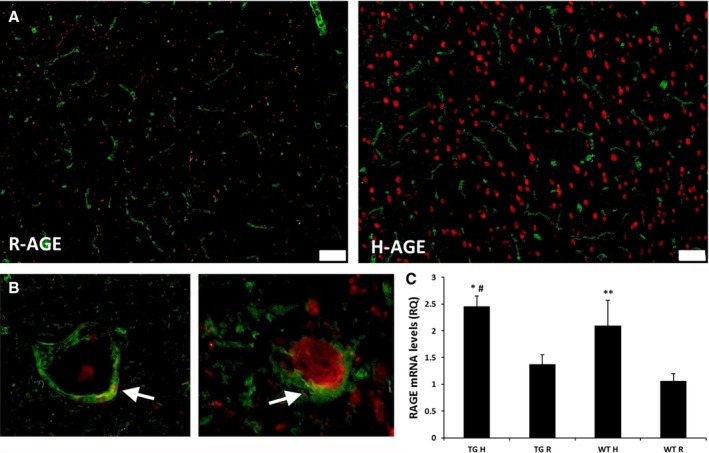
The effect of AGE *due to irradiated diet* on RAGE expression in the brain. (A) Representative figure of RAGE expression in the cortex. AGE receptors were increased in the cortex of high‐AGE diet mice (both Tg2576 and WT). Red, anti‐RAGE antibody staining, green, blood vessels stained by FITC conjugated fluorescein L. esculentum lectin, an endothelial cell marker. Scale bar, 50 μm. (B) RAGE expression on the capillaries was increased in H‐AGE diet mice compared to R‐AGE diet. (C) RAGE mRNA level was upregulated by AGE diet. Tg‐H *P < 0.05 vs. Tg‐R, and ^#^ *p* < 0.05 vs. WT‐R; **p < 0.05 WT‐H vs. WT‐R; Data are mean ± SEM,* n* = 5. Scale bar 50 μm.

As expected (Yan *et al*., 2009), expression of RAGE in the frontal cortex of 11 months old mice fed with H‐AGE diet was increased compared with R‐AGE diet (Fig. 4A). Similarly, RAGE expression was increased in brain capillaries of the H‐AGE diet groups (Fig. 4B). RT–PCR shows RAGE mRNA level was upregulated by AGEs due to irradiation diet. Tg‐H *P < 0.05 vs. Tg‐R and #*P* < 0.05 vs. WT‐R; WT‐H **P < 0.05 vs. WT‐R (Fig. 4C).

#### Levels of AGEs in hippocampus

**Figure 5:**
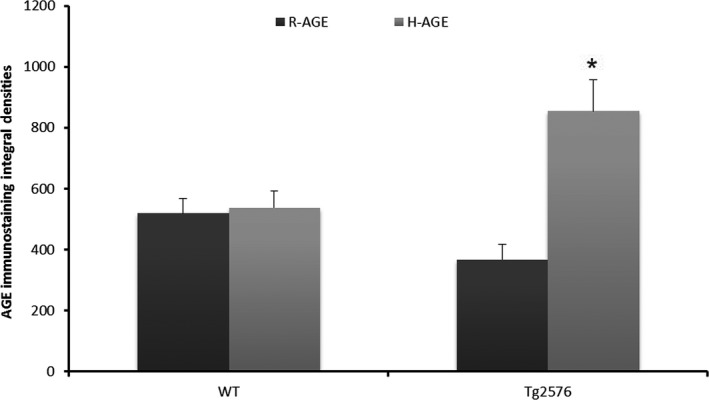
Dot blot analysis was used to analyze insoluble Aβ fraction extracted from hippocampus, immunoprobed with anti‐AGE antibody. Two microliters of protein lysates was spotted on membrane. Data are integral densities with subtracted background, corrected for protein concentration (Mean ± SEM) *P < 0.001 vs. Tg2576 on R‐AGE diet and WT on both diets, *n* = 8 per group.

As there is evidence for co‐localization of AGEs in AD neurotic plaques (Wong *et al*., 2001), we further investigated the involvement of AGEs due to irradiation diet in Aβ aggregation. Levels of AGEs in brain tissue were measured by dot blot. AGEs were significantly higher in hippocampal Aβ insoluble fraction of Tg2576 mice on H‐AGE diet compared to R‐AGE diet and to WT mice (Fig. 5).

## Discussion

Aβ aggregation is a hallmark of the pathogenesis of AD. However, the cause for this aggregation is still unclear. Although accumulation of AGEs is a normal feature of aging (Luth *et al*., 2005), in AD, it is accelerated (West *et al*., 2014). AGEs modification may explain many of the neuropathological and biochemical features of AD such as extensive protein cross‐linking, oxidative stress, and neuronal cell death. *In vivo* studies found that AGEs are co‐localized with protein deposits such as Aβ plaques in AD (Choei *et al*., 2004; Krautwald & Munch, 2010).

In this study, we found that Tg2576 mice on H‐AGE diet had spatial learning impairment as tested by the MWM test (Fig. 2A) further supporting the notion of learning and memory impairment due to a irradiation diet rich with AGEs. The learning curve of WT mice was not affected by H‐AGE diet as reflected in similar memory and learning performances in the acquisition phase to those of WT mice on R‐AGE diet. This suggests higher vulnerability of brains susceptible to AD neuropathology to high dietary AGEs levels consistent with findings of co‐localization of AGEs with neuritic plaques in human AD brains (Wong *et al*., 2001; Cai *et al*., 2014).

It is important to note that due to the violent behavior which characterizes Tg2576 mice, all mice (WT and Tg2576) were individually housed. This social isolation may have had an impact on cognitive performance of otherwise intact mice. It is possible that the failure of WT mice on R‐AGEs diet to perform as expected is a result of the social isolation, and the lack of differences between diets in WT mice is actually a manifestation of a ‘floor effect’. Tg2576 mice on the other hand may have ‘more room’ to deteriorate mainly due to the extent of neuropathology.

Oxidative damage to the vasculature (i.e., aorta) was increased in both WT and Tg2576 mice reflecting overall deleterious effects of dietary AGEs. This increase was highest in the Tg2576 group (10 times higher in mice fed H‐AGE diet compared to a regular diet). Importantly, in the Tg2576 H‐AGE diet group, MG and CML were highly correlated with hippocampal Aβ42.

These results also demonstrate that irradiation food rich in AGEs seem to have systemic and central effects and are also consistent with prior evidence indicating that AGEs intake and accumulation play a central role in vascular damage (Southern *et al*., 2007). As peripheral oxidative stress predicts AD (Berr *et al*., 2000; Torres *et al*., 2011), our results may suggest that oxidative damage to the vasculature caused by dietary AGEs is associated with Aβ aggregation in the brain.

Receptor for advanced glycation end product expression is highly upregulated in cerebral vessels, microglia, and neurons in AD human and rodent brains (Zlokovic, 2008). In addition, increasing levels of RAGE has been suggested as a cause for amyloid aggregation in the aging and AD brains (Arancio *et al*., 2004; Chaney *et al*., 2005). Fig. 4 shows upregulation of RAGE expression in frontal cortex is associated with a high AGEs intake and a similar association for cerebral capillaries, suggesting that AGEs intake and accumulation may play a significant role in vascular damage and enhance plaque formation in the brain. Although it is not yet clear which species of Aβ (soluble or insoluble) binds to RAGE (Yan *et al*., 2009), the strong increase of RAGE in the high‐AGE diet group together with the significant correlation of insoluble Aβ_42_ with AGEs intake suggests that insoluble Aβ binds to RAGE. This is consistent with the influx of Aβ across the BBB depending on its interaction with RAGE (Mackic *et al*., 1998) and that it was shown that AGEs increase the amount of fibronectin in pericytes and directly induce hypertrophy at the BBB via RAGE signaling (Shimizu *et al*., 2013). In addition, levels of AGEs were significantly higher (~twofold) in hippocampal insoluble Aβ fraction in the Tg2576 group on H‐AGE diet compared to the other three groups as shown in Fig. 5, suggesting that exogenous AGEs pass the BBB and may potentiate Aβ aggregation in line with evidence of AGE surrounding mature amyloid plaques in the AD brains (Wong *et al*., 2001).

Cai *et al*., 2014 showed that cognitive dysfunction develops in parallel with metabolic changes in old WT mice fed defined MG diet and that deposits of AGEs and Aβ in brains of such mice were increased. In addition, MG diet induced metabolic syndrome which in turn has been associated with increased risk of AD and dementia (Gorospe & Dave, 2007; Raffaitin *et al*., 2009). This is consistent with our findings of an association of weight gain, in the context of H‐AGE diet, with increased insoluble Aβ_42_. Supported by clinical findings (Beeri *et al*., 2011), these results further validate the relevance of dietary AGEs as a modifiable risk factor for AD in humans. Urine and serum levels of AGEs were increased in H‐AGE diet.

Our study has limitations. The observed biological effects were primarily interpreted as a consequence of high‐AGE content of the irradiated diet, focusing on MG and CML as both modifications can engage RAGE and trigger proinflammatory effects (Arancio *et al*., 2004; Chaney *et al*., 2005). However, a number of other AGEs and oxidative changes to food constituents, not assayed in this study, might have contributed to the findings. These potentially include other AGEs derivatives of glucose–protein such as pentosidine that were shown as indicators for AD (Meli *et al*., 2002), oxidative damage to tryptophan and methionine residues. However, it is important to note that evidence regarding the effects of irradiation on nutrients is controversial. Irradiation had no effect on protein digestibility, biological value or net protein utilization (Mossel & De Groot, 1964), on protein quality and little effect on amino acid content (Ley *et al*. 1969), or on total protein; however, the solubility of total fraction has been shown to significantly decrease (Afify *et al*., 2011). Similarly, lysine content, the most critical amino acid in poultry nutrition, was unchanged after irradiation at 25 kGy (Landolt, 1982). In contrast, there is evidence for loss of some fat‐soluble vitamins due to irradiation (Coates *et al*., 1969). Although the potential for lipid peroxidation exists, the degree to which this occurs in the irradiation diet [with relatively low fat content (6.2%) and where the most unsaturated fatty acid in linolenic acid (C18:3)] is minimal and not at a level that would adversely affect intake.

The irradiated diet increased the levels of AGEs affecting minimally other components. Finally, although we measured fasting glucose, we did not measure insulin levels, and recently, low AGE diet has been linked to improved insulin sensitivity in diabetes.

As diet is a modifiable lifestyle, restricting AGEs consumption by cooking at lower temperatures may be a simple noninvasive intervention that has potential of reducing or preventing the devastating symptoms of AD.

## Experimental procedures

### Dietary formulas

Teklad Global Rodent Diet 2918 (Harlan Laboratories, Jerusalem, Israel) is an irradiated diet and is fortified with supplements to offset irradiated‐depleted micronutrients (Table 3). In this study, we used two forms of this standard 2018 and 2918 Rodent Diet; one form was prepared without exposure to irradiation (2018), and the second form consisted of the same chow exposed to irradiation (exposed to gamma irradiation from a cobalt‐60 source) as per standard procedure. The dietary formulas were nutritionally equivalent, differing in AGEs content, based on assessment of CML‐BSA and MG derivatives by ELISA, using monoclonal anti‐CML (STA ‐316; Cell Biolabs, Inc, San Diego, CA, USA) and anti–MG (STA‐311). The irradiated standard formula contained 5.1 ng of CML and 14.5 ng of MG derivatives per g protein, herein termed high‐AGE diet (H‐AGE); the formula prepared in the absence of exposure to irradiation contained threefold lower levels of CML (1.6 ng g^−1^) and 2.3‐fold lower MG derivatives (6.2 ng g^−1^), termed Regular‐AGEs diet (R‐AGE; Table 3). Both dietary formulas were pelleted by the manufacturer and kept at room temperature.

**Table 3:** Characteristics of Dietary Formulas

Nutrients	R‐AGE	H‐AGE
Protein (%)	18.6	18.6
Fat (%)	6.2	6.2
Carbohydrates (%)	44.2	44.2
Energy density (kcal/g)	3.1	3.1
MG (ng/g)	6.2	14.5
CML (ng/g)	1.6	5.1

### Animals

For this experiment, we used 20 C57BL/6J wild‐type (WT) and 20 Tg2576 male mice. Briefly, male Tg2576 mice, which express human APP with the ‘Swedish’ mutation, were purchased from Taconic (catalog #001349) at the age of 2 months and self‐bred with C57BL/6J females in our laboratory (on C57BL/6J background). Half of the animals from each strain were fed normal rodent chow and water *ad libitum*; the others were given *ad libitum* access to the irradiated diet. Animals were housed one per cage in a temperature‐controlled room with a 12 h light–dark cycle and provided with food and water *ad libitum*. Animals were weighed monthly and maintained on their respective diets for 7 months. Baseline body weights and urine samples were obtained, and mice were randomly assigned to either H‐AGE diet [WT (*n *=* *10) and Tg2576 (*n *=* *10)] or R‐AGE diet [WT (*n *=* *10) and Tg2576 (*n *=* *10)] for up to 44 weeks of age. Food and water intake were recorded daily for 1 week and biweekly thereafter. Then, they were assessed for memory function and AD‐like neuropathology at 11 months of age. Mice were anesthetized with the general inhalation anesthetic 1‐chloro‐2,2,2‐trifluoroethyldifluoromethyl ether (isoflurane; Baxter Healthcare Corp., Deerfield, Israel) and sacrificed by decapitation. Brain specimens were harvested and hemi‐dissected: one hemisphere was fresh‐frozen for histological/morphological studies. Frontal and parietal cortices, hippocampus, and cerebellum were dissected from the other hemisphere. Specimens were rapidly frozen, pulverized in liquid nitrogen, and stored at −80 °C for subsequent analysis of Aβ_42_ peptide. All procedures were approved by the Institutional Animal Care and Use Committee of the Sheba Medical Center.

#### RAGE immunofluorescence

Brain slices were fixed with 4% paraformaldehyde rinsed in PBS [0.1 m (pH 7.2)]. The slices were permeabilized and blocked with 0.1% Triton X‐100/PBS (PBST) containing 10% normal serum to reduce nonspecific adherence of antibodies. Brain slices were incubated in primary antisera (rabbit anti‐RAGE, 1:1000; Chemicon, Temecula, CA, USA) for 1 h at 25 °C in a humid chamber. After incubation with the primary antisera, the slices were rinsed with PBST three times and incubated with anti‐rabbit Alexa Fluor‐568 conjugated secondary antibody (Molecular Probes). Slices were rinsed with PBST three times and incubated with fluorescein (tomato) lectin (1:100 Vector Laboratories) for 1 h at room temperature. Sections were then rinsed three times with PBST and coverslipped with fluoromount mounting medium (Sigma, Rehovot, Israel).

#### RT–PCR for RAGE

Total mRNA was extracted from brain using RNeasy micro kit (QIAGEN, Hiden, Germany) according to the manufacturer's instructions. Reverse transcription of 2 μg of total mRNA was conducted using the high‐capacity cDNA reverse transcription kit (Applied Biosystems, Rhenium Ltd., Modi'in, Israel). Expression of the RAGE and glyceraldehydes‐3‐phosphate dehydrogenase product (GAPDH) was determined by real‐time PCR using Applied biosystems step one plus system with Taqman Gene Expression Assays (Roche Diagnostic, Mannheim, Germany). All real‐time reactions (unknown samples, and controls) were performed in duplicate. The relative expression levels were calculated by normalizing the targets to the endogenously expressed housekeeping gene (GAPDH).

#### Measurement of superoxide formation using dihydroethidium (DHE) in aorta

Dihydroethidium, an oxidative fluorescent dye, was used to detect superoxide in segments of aorta as described previously (Dayal *et al*., 2002). Briefly, fresh unfixed segments of the common aorta were frozen in OCT compound, and transverse sections (10 μm) were generated with a cryostat and placed on glass slides. Sections were then incubated in a light‐protected chamber at room temperature for 20 min. Stock DHE was dissolved at a concentration of 10 mg mL^−1^ in DMSO, diluted to 150 μm in PBS‐buffered saline solution [10 mm PBS (pH 7.4)] immediately before use, and then added directly on the slide at a 1:1000 dilution, giving a final concentration of 3 μm with 10 μmol L^−1^ DHE (Molecular Probes). The sections were observed with 20× magnification under aBX‐43 fluorescence microscope (Olympus, Tokyo, Japan). Color pictures were acquired and analyzed using a digital camera system coupled to an imaging software (Cellsens Entry digital imaging software; Olympus) under a constant exposure time, gain, and offset, chosen as to increase the threshold for fluorescence.

#### AGEs in urine and serum

Urine was collected for 24 h in metabolic cages at baseline and at 11 months. Total protein was assessed by colorimetric detection (Thermo Scientific Pierce BCA Protein Assay Kit Rockford, IL, USA). The urine CML/MG content was measured by competitive enzyme‐linked immunosorbent according to manufactory protocol (Cell Biolabs, Inc., San Diego, CA, USA) assay and expressed as a ratio relative to protein concentrations in 24 h (mg/24 h) in urine.

Serum AGEs were measured by ELISA: AGE levels were measured by competitive ELISAs using monoclonal anti‐CML antibody (4G9) or anti‐MG antibody (3D11) (Mitsuhashi *et al*., 1997; Cai *et al*., 2002). Briefly, 96‐well ELISA plates coated with AGE‐BSA or MG‐BSA overnight were washed three times with washing buffer and then blocked with 150 μL of SuperBlockTM blocking buffer at RT for 1 h. After three rinses with washing buffer, 50 μL of competing antigen was added, followed by 50 μL of either anti‐CML antibody or anti‐MG antibody in dilution buffer containing 2% NGS. Plates were incubated at room temperature for 2 h. Wells were then rinsed three times with washing buffer. Secondary antibody in dilution buffer with 1% NGS was then added to each well and the plates were incubated at 37 °C for 1 h. After rinsing three times, 100 μL of pNPP substrate was added to each well. Optical density (OD) at 405 nm was determined by an ELISA reader. AGE values were expressed as CML units mL^−1^ or MG nmol mL^−1^.

#### Measurement of hippocampal Aβ

For quantitative assessment of hippocampal formation Aβ peptides, frozen pulverized tissue was extracted in a two‐step extraction. The tissue was homogenized in 1% Triton X‐100, diluted in 25 mm phosphate‐buffered saline containing 137 mm NaCl and protease inhibitors cocktail (Roche Biochemicals, Indianapolis, IN, USA) and centrifuged for 1 h at 4 °C at 100 000 *g*. The fraction was designated Aβ_sol_. The remaining pellet was then sonicated in 5m guanidine HCl with 50 mm Tris PH = 8 and protease inhibitors cocktail, incubated for 2 h at 25 °C, and centrifuged at 13 000 *g* for 20 min at 4 °C. The latter fraction was designated Aβ_insol_. Aβ1‐42 was measured by sandwich ELISA (WAKO, Osaka, Japan) according to manufacturer's instructions.

#### AGEs in hippocampus

For the dot blot assays, 2 μL of the insoluble fraction of hippocampal Aβ extract was spotted on nitrocellulose (Bio‐Rad, Prague, Czech Republic), and the membrane was dried at room temperature for 30 min and then blocked with Roti‐Block (Carl Roth, Karlsruhe, Germany) 1:10 with distilled H_2_O for 1 h and probed with anti‐AGEs antibody (KH001‐02, peroxidase conjug.; 1:200 dilution; Trans Genic, Inc.; monoclonal clone 6D12, Fukuoka, Japan) overnight. After washing (3× with PBS/0.1% Tween‐20), visualization of the dots was performed with diaminobenzidine staining [10 mg of 3,3‐DAB (Sigma)] in 20 ml PBS containing 10 μl of 30% hydrogen peroxide (Sigma). The blots were scanned using Molecular Imager ChemiDoc XRS+ (Bio‐Rad), and quantification of spot integral optical densities was calculated by the imagej software (NIH Image). Data are integral densities with subtracted background, corrected for protein concentration.

#### Behavioral testing

##### Open field

The open field apparatus was a square field (50 × 50 × 30 cm) made of white acrylic material. Each 10 month mouse was placed in the corner of the apparatus/open field at the beginning of the test and allowed to move freely as single exploration trial for 5 min. The floor and walls of the field were cleaned thoroughly with 70% ethanol and air‐dried after each trial to remove olfactory cues. The total distance and total center time were recorded using a ceiling‐mounted video camera (Tracker VP200; HVS Image, Hampton, UK) for later analysis. The image of the field was divided into 16 equally sized squares (FOUR inner and 12 outer).

The total distance (= total path), the % of time moving, % of cells used was evaluated as an index of locomotor activity, and the % time spent in the center cells was evaluated as an index of anxiety. The data analysis was performed using hvs image field 2100 software, Hampton, UK.

##### Morris water maze (MWM)

The spatial reference learning and memory of all mice were assessed using the MWM. Mice were tested in a 140 cm circular pool filled with water at 25–27 °C and made opaque by the addition of nontoxic white acrylic paint. In a prelearning phase, mice were trained to mount a submerged escape platform (20′20 cm) in a restricted region of the pool (contained by a circular vinyl insert 60 cm in diameter). For the subsequent trials, the pool was divided into four quadrants. The four start positions were located at the intersections of the quadrants. A platform was hidden 1.5 cm below the water level. The mice were first placed into the water facing the wall of the pool and allowed to swim until it located and climbed onto the submerged platform. Each mouse received four trials per day for six consecutive days of the learning phase. The start position was changed in each trial. The swim time to reach the platform was recorded in each trial. Mice failing to reach the platform in 60 s were led to the platform and placed on the platform for 15 s. On the 7th day, in the probe trial, the platform was removed from the pool and mice were allowed to search for the platform for 60 s. All trials were monitored by a camera above the pool. The time spent searching for the platform and the target quadrant frequency were recorded. Prior to each entry into the water maze pool, mice were placed in a thermoregulated holding chamber filled with water (3 cm deep) for 90 s, so that anxiety responses (related to experimenter handling and exposure to water) could be minimized. To control for potential hypothermia, following each trial, mice were towel‐dried and returned to a prewarmed home cage. Water maze activities were monitored with the HVS image 2100 Plus Track video tracking system (Buckingham, UK). Mice were tested during the second and third hours of the night portion of the light cycle. All animals were individually coded, and investigators were blind to group designations throughout testing (Vorhees & Williams, 2006).

#### Statistics

Results are expressed as means + SEM. Differences between mean values were determined using the Student *t*‐test, repeated‐measures anova using the Huynh–Feldt adjustment, two‐way anova, or one‐way anova when appropriate, according to the experimental design. A *P*‐value of 0.05 was considered significant. All significance tests were two‐sided. SPSS version 20.0 (IBM corporation, NY, USA) was used for data analyses.

## Funding

No funding information provided.

## Conflict of interest

The authors declare no competing financial interests.

## Author Contributions

I Lubitz wrote the manuscript; M Beeri brought funding for the study and supervised manuscript writing; I Lubitz, J Ricny, D Atrakchi‐Baranes, C Shemesh, S Liraz‐Zaltsman, A Maksin‐Matveev, A Leibowitz, and Weijing Cai preformed the experiments; J Schmeidler and E Kravitz performed data analyses; and I Cooper, Z Kristofikova, D Ripova, D Leroith, J Uribarri, and J Schmeidler reviewed/edited the manuscript.
